# Activated neutrophils induce an hMSH2-dependent G2/M checkpoint arrest and replication errors at a (CA)13-repeat in colon epithelial cells

**DOI:** 10.1136/gut.2007.141556

**Published:** 2008-02-13

**Authors:** C Campregher, M G Luciani, C Gasche

**Affiliations:** Medical University of Vienna, Department of Internal Medicine III, Division of Gastroenterology and Hepatology and Christian Doppler Laboratory on Molecular Cancer Chemoprevention, Vienna, Austria

## Abstract

**Objective:**

Chronic inflammation in ulcerative colitis is associated with increased risk for colorectal cancer. Its molecular pathway of cancer development is poorly understood. We investigated the role of neutrophil-derived cellular stress in an in vitro model of neutrophils as effectors and colon epithelial cells as targets, and tested for changes in cell cycle distribution and the appearance of replication errors.

**Design:**

Colon epithelial cells with different mismatch repair phenotypes were co-cultured with activated neutrophils. Target cells were analysed for cell cycle distribution and replication errors by flow cytometry. Changes in nuclear and DNA-bound levels of mismatch repair- and checkpoint-related proteins were analysed by western blotting.

**Results:**

Activated neutrophils cause an accumulation of target cells in G2/M, consistent with the installation of a DNA-damage checkpoint. Cells that do not express hMSH2, p53 or p21^waf1/cip1^ failed to undergo the G2/M arrest. Phosphorylation of p53 at site Ser15 and Chk1 at Ser317, as well as accumulation of p21^waf1/cip1^, was observed within 8–24 h. Superoxide dismutase and catalase were unable to overcome this G2/M arrest, possibly indicating that neutrophil products other than superoxide or H_2_O_2_ are involved in this cellular response. Finally, exposure to activated neutrophils increased the number of replication errors.

**Conclusions:**

By using an in vitro co-culture model that mimics intestinal inflammation in ulcerative colitis, we provide molecular evidence for an hMSH2-dependent G2/M checkpoint arrest and for the presence of replication errors.

Chronic inflammation leads to tumour development.^1^ Ulcerative colitis is associated with an increased risk of development of colorectal carcinoma (CRC). One of the key features of ulcerative colitis is the presence of crypt abscesses, which are accumulations of polymorphonuclear cells (PMNs) within colonic crypts.^2^ ^3^ It has been suggested that reactive oxygen species (ROS) released by PMNs are one of the main contributing factors to colon carcinogenesis.^1^ Oxidative stress can alter cellular components including proteins, mRNAs and DNA.^4^^–^6 It is unclear, however, whether oxidative stress on its own may cause mutations in cells.^7^ ^8^ Activated PMNs not only produce ROS, but also excrete lactoferrin^9^ and other proteins including several cytokines.^10^ ^11^ Thus, previous in vitro studies that focused on H_2_O_2_-induced mutagenesis^8^ ^12^ only partially reflected the pathophysiological condition of colon carcinogenesis.

The mismatch repair (MMR) system plays a central role in promoting genetic stability by correcting DNA replication errors. Homologs of the bacterial MutS and MutL MMR proteins in eukaryotes form heterodimers with discrete roles in MMR-related processes. The discovery of a link between human cancer and MMR defects has led to an increased interest in eukaryotic MMR.^13^ Frameshift mutations of short-tandem repetitive sequences indicate instability of these sequences [microsatellite instability (MSI)] and represent a hallmark of MMR deficiency in human cancers.^14^ ^15^ Since MSI can be detected in colitis tissue without dysplasia, inactivation of the MMR system must be an early event in colon carcinogenesis in ulcerative colitis. However, the nature of inflammation-induced microsatellite mutations is still obscure. The MMR system can be activated after replication to repair DNA errors. Evidence suggested that the proliferating cell nuclear antigen (PCNA) is required for MMR recruitment prior to DNA repair synthesis,^16^ leading to the hypothesis that replication and MMR may be coupled and that the replication fork provides the strand discrimination signal for repair.^17^

Exposure of eukaryotic cells to agents that alter the DNA structure results in transient arrest of the progression through the cell cycle. Ataxia telangiectasia mutated kinase (ATM) acts as a sensor of oxidative damage, coordinating stress responses with cell cycle checkpoint control and repair of such damage.^18^ Cell cycle checkpoints give the cell the opportunity to either mend the DNA damage or undergo apoptosis. In particular, the G2/M checkpoint allows cells to overcome replication errors before entering mitosis, thereby ensuring genomic integrity. Apart from ATM, key components of the G2/M cell cycle checkpoint include the ATM-and-Rad3-related kinase (ATR), the downstream checkpoint kinases Chk1 and Chk2^19^ ^20^ and the tumour suppressor protein p53,^21^ which is stabilised by phosphorylation at ATM and ATR sites.^22^ ^23^ Phosphorylation of p53 correlates with enhanced transcription of the cyclin-dependent kinase inhibitor p21^waf1/cip1^.^24^ ^25^ DNA-alkylating agents induce phosphorylation and activation of p53, leading to an increased expression of p21^waf1/cip1^. Cell lines with MMR deficiency are resistant to these alkylating agents and bypass the cell cycle arrest, indicating that the MMR has a role in post-replication checkpoints.^26^ ^27^ However, nitric oxide (NO) and H_2_O_2_ are capable of arresting hMLH1 mutant cells in G2/M.^4^ ^28^ No information exists on the role of hMSH2 in mediating such a cell cycle arrest.

In this work, we hypothesise that the chronic exposure of the intestinal mucosa to activated PMNs leads to DNA damage, which may activate checkpoint kinases and initiate MMR, or if this is inefficient, may drive colon carcinogenesis. In order to simulate the carcinogenic environment in ulcerative colitis, we established an in vitro co-culture system with primary PMNs as effector cells and various colon cell lines as targets. Our results show that exposure of colon cells to activated PMNs install a G2/M cell cycle checkpoint, indicative of DNA damage, through a mechanism that does not require hMLH1, but rather p53/p21 and hMSH2. This G2/M arrest is associated with an increase in replication errors.

## MATERIALS AND METHODS

### Cell lines

The human colorectal carcinoma cell lines HCT116^hMLH1−/−^ and their derivatives HCT116+chr3^hMLH1+/−^,^26^ HCT116+chr3 A3.1, HCT116+chr3 A3.7,^29^ HCT116-mlh1-2^hMLH1+/−^, HCT116^p53−/−^, HCT116^p21−/−^30 and Lovo^hMSH2−/−^ and their derivatives Lovo+chr2^hMSH2+/−^ Lovo(DT40.2)-4-1^hMSH2−/−^,^31^ as well as the human endometrial adenocarcinoma cell line HEC59^hMSH2−/−^ and HEC59+chr2^hMSH2+/−^32 were grown in IMDM (Gibco/Invitrogen, Lofer, Austria)  containing 10% fetal bovine serum (FBS; Biochrom, Berlin, Germany). The medium for HCT116+chr3 contained 400 μg/ml and for HEC59+chr2 and Lovo+chr2 cells 700 μg/ml G418 (Gibco), respectively. The medium for HCT116-mlh1-2 cells^33^ contained 100 μg/ml hygromycin B (Invitrogen). The clones HCT116+chr3 A3.1 and HCT116+chr3 A3.7 were grown with 150 μg/ml hygromycin B and 400 μg/ml G418. The promyelocytic leukaemia cells line HL60 (ATCC CCL-240) was cultured in RPMI (Gibco/Invitrogen) supplemented with 10% FBS.

### Isolation and activation of polymorphonuclear cells

PMNs were freshly isolated from heparinised blood of healthy volunteers by dextran T500 (Pharmacia, Uppsala, Sweden) sedimentation followed by density gradient centrifugation through Ficoll-Paque (Amersham, Uppsala, Sweden) or HL60 cells were derived to granulocyte-like neutrophils by differentiation as described previously;^34^ erythrocytes were lysed in NaCl (0.2%) followed by NaCl (1.6%) and cells were washed in Ca/Mg-free Hanks’ balanced salt solution (HBSS) (Gibco). CD66b mAb (BD 55572) was used to confirm the purity of isolated PMNs by flow cytometry.^35^ PMNs were activated in HBSS containing 50 ng/ml phorbol 12-myristate 13-acetate (PMA; Sigma, St Louis, Missouri, USA) at 37°C and 5% CO_2_ for 30 min. PMA was removed by washing the PMNs twice with HBSS. The production of ·O_2_^−^ was determined by lucigenin-enhanced chemiluminescence as described.^36^ ^37^ Superoxide dismutase (SOD, 1000 U/ml) (Sigma) and catalase (CAT, 1000 U/ml) (Sigma) were used as scavengers for ·O_2_^−^ and H_2_O_2_, respectively.

### Co-culture and cell cycle analysis

Target cells at a concentration of 7×10^4^ were seeded onto 6-well plates. Twenty-four hours later, PMNs were added to the upper chamber of a Transwell 0.45 μm microporous insert preventing cell-to-cell contact. PMNs and target cells were co-cultured at effector:target ratios of 0:1 to 100:1 for up to 24 h. Target cells were harvested using accutase (PAA Laboratories, Linz, Austria), fixed and analysed for cell cycle distribution as described.^36^

### Western blot analysis

Cell lysates and DNA-bound fractions were obtained as described.^36^ For western blotting, 50–150 μg of lysates were used with the following antibodies: rabbit polyclonal antibody (pAb) anti-phospho-p53 Ser15 (Cell Signaling Technology, Danvers, MA, USA); pAb anti-cleaved caspase-7 (Cell Signaling); mAb anti-p21^waf1/cip1^ (Cell Signaling); mAb anti-hMSH2 (Becton-Dickinson, San Jose, California, USA), mAb anti-hMHS6 (Becton-Dickinson), mAb anti-hPMS2 (Becton-Dickinson), mAb anti-hMre11 (Becton-Dickinson) and mAb anti-hMLH1 (Becton-Dickinson); mAb anti-tubulin (Abcam); mAb anti-actin (Sigma); pAb anti-phospho-Chk1 Ser317 (Cell Signaling).

### Analysis of replication errors

Five thousand non-fluorescent HCT116+ch3 cells, bearing the enhanced green fluorescent protein (EGFP)-based plasmid  pIREShyg2-EGFP/CA13 (clones A3.1, one plasmid copy, and A3.7, two plasmid copies)^29^ were sorted on a FACSVantage SE using CloneCyt Plus sorting technology (Becton Dickinson Immunocytometry Systems), and PMNs were activated with PMA as described above. Cells were cultured at 100:1 ratios for 24 h and PMNs were removed. Target cells were grown for an additional 7 days. The EGFP-positive population with low fluorescence intensity was considered as the “transiently mutated fraction” (M1), and that with high fluorescence intensity as the “permanently mutated fraction” (M2).^29^

### Statistical analysis

Experiments were carried out at least in triplicates and repeated twice. Data are represented as mean with the SD and compared by using the Student t test. p-Values <0.05 were considered to be statistically significant.

## RESULTS

### Establishment of an in vitro co-culture system

**Figure 1:**
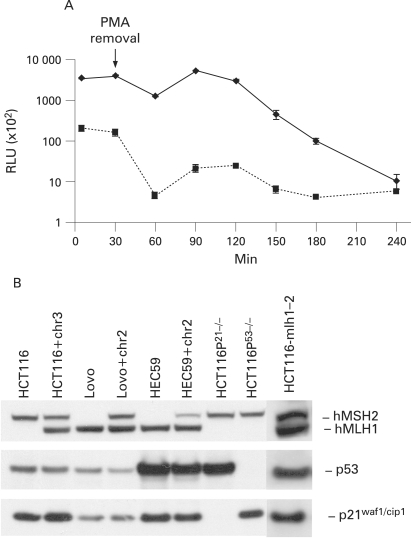
Characterisation and analysis of colon epithelial cell lines and ·O_2_^−^ release by activated polymorphonuclear cells (PMNs). (A) Freshly isolated PMNs were activated with 50 ng/ml phorbol 12-myristate 13-acetate (PMA) for 30 min. Cells were washed twice and ·O_2_^−^ release was measured by lucigenin-amplified chemiluminescence. A strong induction of ·O_2_^−^ release was observed upon activated PMNs (solid line) that lasted for more than 2 h. Data are given as relative light units (RLUs) per 1×10^6^ cells. Non-activated cells (dotted line) were subjected to the same procedures (isolation and washing) and served as a control. A slightly increased level of ·O_2_^−^ release by non-activated cells was observed. Each data point presents the mean (SD) of three independent measurements. (B) Western blotting of hMLH1, hMSH2, p21^waf1/cip1^ and p53 expression in target cell lines. The western blot confirmed the expected loss or reinstalled expression of protein expression of these cells (HCT116^hMLH1−/−^, HCT116+chr3^hMLH1+/−^, Lovo^hMSH2−/−^, Lovo+chr2^hMSH2+/−^, HEC59^hMSH2−/−^, HEC59+chr2^hMSH2+/−^, HCT116^p21−/−^, HCT116^p53−/−^ and HCT116-mlh1-2).

To simulate the carcinogenic environment in ulcerative colitis, we established an in vitro co-culture system in which PMNs were co-cultured with various colon epithelial cells separated by a semi-permeable membrane. Activation of PMNs with PMA is followed by oxidative burst.^38^ A strong and sustained ·O_2_^−^ release was observed for at least 2 h after PMA removal (fig 1A). The expression or absence of MMR components, p53 and p21 was tested (fig 1B) in the various cell lines used in this work.

### Activated polymorphonuclear cells cause an hMHS2-dependent G2/M arrest in colon epithelial cells

**Figure 2:**
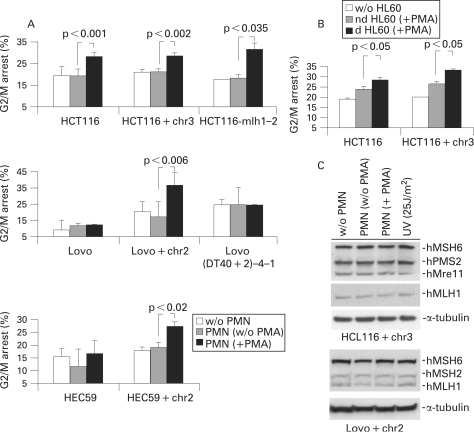
Polymorphonuclear cells (PMNs) cause a G2/M arrest in hMSH2-expressing colon epithelial cells without affecting the expression levels or DNA binding activity of mismatch repair (MMR) proteins. (A) HCT116, HCT116+chr3, HCT116-mlh1-2, Lovo, Lovo+chr2, Lovo(DT40+2)-4-1, HEC59 and HEC59+chr2 cells were co-cultured at a ratio of 20:1 with non-activated or activated PMNs for 24 h and cell cycle distribution was analysed by flow cytometry. A significant increase of the G2/M population was observed in all cell lines upon co-culture with activated PMNs except for Lovo, Lovo(DT40+2)-4-1 and HEC59 (cells lacking wild-type hMSH2). Each column presents the mean (SD) of at least three experiments. (B) HCT116 and HCT116+chr3 cells were co-cultured at 20:1 ratios with non-differentiated (nd) or dimethyl sulfoxide (DMSO)-differentiated (d) HL60 cells, both treated with 100 ng/ml phorbol 12-myristate 13-acetate (PMA). A significant increase of the G2/M population was observed with DMSO-differentiated HL60 cells. Each column presents the mean (SD) of at least three experiments. (C) HCT116+chr3 and Lovo+chr2 cells were co-cultured in the presence of activated PMNs for 24 h and changes in the total protein levels of MMR proteins (hMre11, hMLH1, hPMS2, hMSH2 and hMSH6) were analysed by western blotting. None of the investigated proteins showed a change in expression levels under these conditions; α-tubulin served as a loading control.

**Figure 3:**
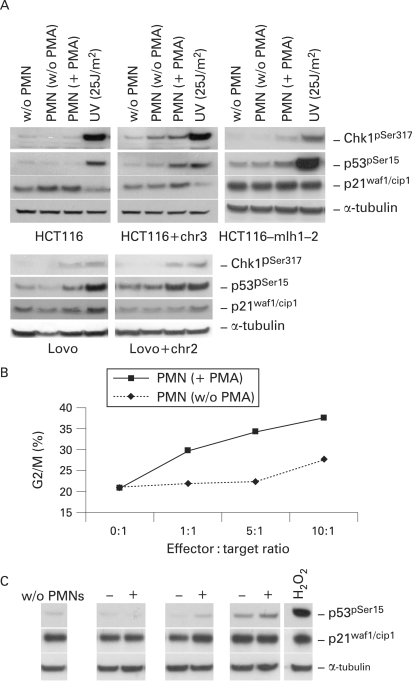
Activated polymorphonuclear cells (PMNs) induce a dose-dependent activation of checkpoint components in colon cells. (A) HCT116, HCT116+chr3, HCT116-mlh1-2, Lovo and Lovo+chr2 cells were co-cultured at a ratio of 20:1 for 8 h; cells were harvested and cell lysates were analysed for total levels and phosphorylation status of checkpoint proteins. An increased phosphorylation of Chk1 at Ser317 and p53 Ser15 was detected in HCT116+chr3, HCT116-mlh1-2 and Lovo+chr2 cells and an accumulation of p21^waf1/cip1^ in HCT116 and HCT116+chr3 cells. Ultraviolet light at an intensity of 25 μJ/m^2^ was used as positive control. α-tubulin served as loading control. (B) HCT116 cells were co-cultured at ratios from 1:1 to 10:1 with non-activated or activated PMNs for 24 h and cell cycle distribution was measured by flow cytometry. A dose-dependent increase of G2/M arrest was observed upon co-culture with activated PMNs. (C) Total lysates of cells exposed to activated PMNs (ratios of 1:1 to 10:1) for 24 h were analysed by western blotting. A dose-dependent increase of p53 phosphorylation at the site Ser15 and total levels of p21^waf1/cip1^ was detectable upon co-culture. Co-culture with non-activated PMNs is indicated as (−) and co-culture with activated PMNs is indicated as (+). H_2_O_2_ (200 μmol/l) was used as positive control.  Tubulin was used as a loading control.

Oxidative stress induces cellular checkpoints, leading to cell cycle arrest and preventing mitosis of cells with defective DNA replication.^39^ Activated PMNs were co-cultured with HCT116, HCT116+chr3, Lovo or Lovo+chr2 cells for 24 h. All cell lines, except Lovo, displayed an increase in the G2/M population within 24 h at 20:1 ratios (aPMN:target cells) (fig 2A), consistent with the MMR component hMSH2, but not hMLH1, being involved in the G2/M arrest. No such effect was observed when using non-activated PMNs. A dose effect was observed when HCT116 were cultured at different effector:target ratios (fig 3B). A similar experiment conducted with HEC59 cells or HEC59+chr2, showed a G2/M arrest only in cells, in which an extra chromosome 2 has been introduced, and therefore express hMSH2 (figs 2A and 1B). The same results were observed with dimethylsulfoxide (DMSO)-differentiated neutrophils (d HL60) derived from HL60 cells (fig 2B). Taken together, these results suggest that activated PMNs induce a G2/M arrest, independent of hMLH1 expression but dependent on hMSH2 expression. Moreover, co-culture of Lovo(DT40+2)-4-1 cells (a Lovo+chr2-derived cell line lacking hMSH2 expression^31^) with activated PMNs revealed no G2/M arrest (similar to Lovo and HEC59 cells; fig 2A), consistent with the assumption that the observed G2/M arrest is hMSH2 dependent.

### Activated polymorphonuclear cells do not change the expression of mismatch repair proteins

It was previously suggested that oxidative stress relaxes the MMR system and reduces hMSH6.^40^ ^41^ As hMSH2 is a potential candidate for installation of the G2/M arrest upon exposure to activated PMNs, we tested for changes in the expression levels of MMR proteins in target cells. However, no changes in MMR protein levels were detectable under these conditions (fig 2C).

### Activation of the ATM/ATR targets Chk1 and p53 is associated with the PMN-induced G2/M arrest

It has been previously established that ATM and ATR are required to activate a p53- and Chk1-dependent G2 arrest upon DNA damage.^42^ Upon 8 h co-culture with activated PMNs, phosphorylation of Chk1 at Ser317 and of p53 at Ser15 was detected in all cells but HCT116 and Lovo whereas an accumulation of p21 was only seen in HCT116 and HCT116+chr3 cells (fig 3A). However, at 24 h, a dose-dependent phosphorylation of p53 at Ser15 and expression of the p53-downstream CDK-inhibitor p21^waf1/cip1^ was observed in HCT116 cells, which was paralleled by an increase in G2/M arrest (fig 3B,C). These results indicate the activation of a DNA-damage checkpoint in colon cells independent of hMLH1. To control for a possible role of additional genes transferred through chromosome 3 we also tested HCT116 cells that had been transfected with an hMLH1 construct (HCT116-mlh1-2), expressing wild-type hMLH1 and displaying MMR proficiency.^33^ When co-cultured in the presence of activated PMNs, they exhibited an increase in G2/M, similar to that described for the parental cell line (fig 2A). Western blotting of lysates from HCT116-mlh1-2 cells, co-cultured with activated PMNs, also showed a similar activation of Chk1 and p53 and accumulation of p21^waf1/cip1^ (fig 3A). Taken together, these results suggest that the presence of hMLH1 accelerates the activation of a checkpoint response but is not essential to achieve such.

### The PMN-induced G2/M arrest depends on the expression of p53 and p21

**Figure 4:**
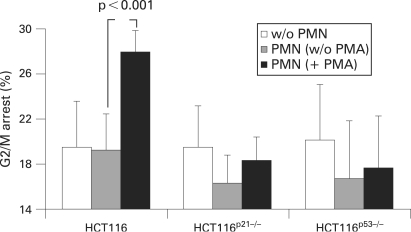
G2/M arrest induced by polymorphonuclear cells (PMNs) is dependent on the expression of p53 and p21^waf1/cip1^. HCT116, HCT116^p21−/−^ and HCT116^p53−/−^ cell lines were cultured with activated PMNs at s ratio of 20:1 for 24 h and analysed for cell cycle distribution by flow cytometry. HCT116^p21−/−^ and HCT116^p53−/−^ cells failed to undergo a G2/M arrest under these conditions. Each column represents the mean (SD) of three independent experiments.

Several reports suggest an essential role for p53 and p21^waf1/cip1^ in installation of the G2/M checkpoint.^30^ ^43^ Indeed, cells in which the p53 or the p21 gene had been disrupted failed to arrest in G2 following gamma ionising radiations.^30^ In fact our experiments demonstrated p53 phosphorylation at Ser15 (an ATM and ATR target site) and p21 accumulation (fig 3A,C). In order to investigate the importance of p53 and p21 in our system, the isogenic cell lines HCT116^p53−/−^ and HCT116^p21−/−^, in which the p53 or p21^waf1/cip1^ genes had been disrupted,^30^ were co-cultured as described above. Both cell lines failed to undergo a G2/M arrest (fig 4), suggesting that the G2/M arrest caused by activated PMNs depends on the expression of both p53 and p21^waf1/cip1^.

### Superoxide dismutase and catalase do not inhibit phosphorylation at p53 Ser15 and increased levels of p21^waf1/cip1^

**Figure 5:**
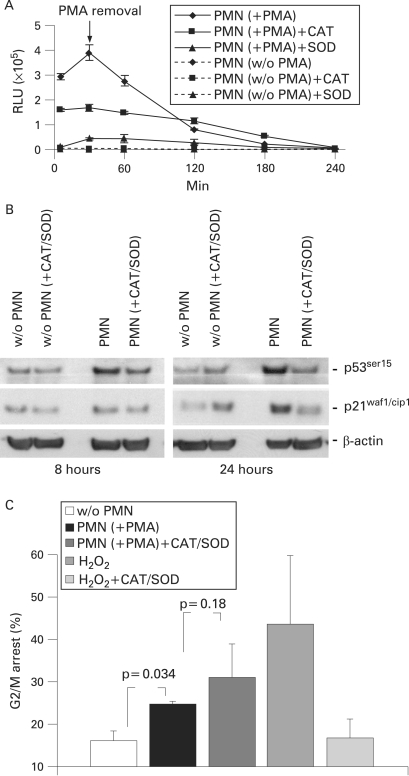
Superoxide dismutase (SOD) and catalase (CAT) do not prevent G2/M arrest but reduce p53 phosphorylation and p21^waf1/cip1^ accumulation. (A) Polymorphonuclear cells (PMNs) were activated with phorbol 12-myristate 13-acetate (PMA) (50 ng/ml) for 30 min. After removal of PMA by washing the cells twice with Hanks’ balanced salt solution (HBSS), ·O_2_^−^ release was measured by lucigenin-amplified chemoluminescence in the presence or absence of SOD (1000 U/ml) or CAT (1000 U/ml). A strong ·O_2_^−^ scavenging effect was observed in the presence of SOD and, to a lower extent, of CAT. Very low ·O_2_^−^ production was measurable in the absence of PMA. (B) HCT116+chr3 cells were co-cultured with activated PMNs in the presence of SOD and CAT as in (A) and the total levels of p21 and p53 phosphorylation at Ser15 were analysed by western blotting. The addition of SOD and CAT considerably reduced both p53 phosphorylation and accumulation of p21^waf1/cip1^ within 8 h. β-Actin served as a loading control. (C) HCT116+chr3 cells were co-cultured as in (A) or exposed to 200 μmol/l H_2_O_2_, in the presence of SOD and CAT, and cell cycle distribution was measured by flow cytometry. Under these conditions, SOD and CAT blocked H_2_O_2_-dependent, but not PMN-induced, G2/M arrest after 24 h.  RLU, relative light units.

SOD catalyses the reduction of ·O_2_^−^ to oxygen and H_2_O_2_, whereas catalase (CAT) catalyses the reduction of H_2_O_2_ to water and oxygen. ·O_2_^−^ release by activated PMNs was measured by lucigenin-amplified chemiluminescence in the presence of SOD, CAT or both enzymes. SOD but not CAT showed a strong ·O_2_^−^ scavenging effect (fig 5A). To test the effect of these enzymes on the G2/M arrest, total cell lysates of HCT116+chr3 cells were analysed following co-culture in the presence of both CAT and SOD. Although the addition of CAT and SOD during co-culture with activated PMNs reduced the phosphorylation of p53 at site Ser15 and accumulation of p21^waf1/cip1^ (fig 5B), it had no effect on the G2/M arrest (fig 5C), suggesting that PMN products in addition to ·O_2_^−^ or H_2_O_2_ activate the p53/p21 pathway and are sufficient for the installment of the cell cycle arrest.

### Activated polymorphonuclear cells cause replication errors in colon epithelial cells

**Figure 6:**
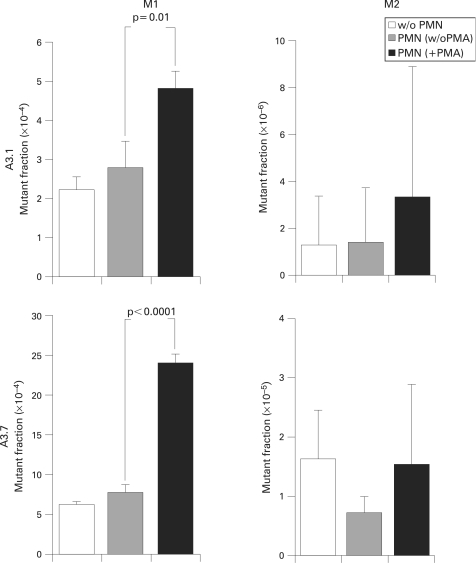
Activated polymorphonuclear cells (PMNs) increase replication errors. (A) HCT116+chr3 cells (clones A3.1 and A3.7 harbouring the pIREShyg2-EGFP/(CA)13 construct) were co-cultured with activated PMNs at a ratio of 100:1 for 24 h. After removal of the PMNs, the target cells were expanded for another 7 days and analysed by flow cytometry. Activated PMNs caused a significant increase in the M1 mutant fraction (ie, transient mutations (replication errors) that may be repaired) but not in the M2 mutant fraction (ie, permanent DNA mutations that are transmitted to daughter cells and may expand) in both clones.

Recruitment of the MMR complex following DNA replication errors leads to cell cycle arrest.^44^ Our experiments so far show an hMSH2-dependent G2/M arrest, which may be a consequence of an increase in replication errors upon exposure to activated PMNs (fig 2A). HCT116+chr3 (clones A3.1 and A3.7) bearing a GFP-expressing plasmid in which the EGFP sequence is kept out of frame by a (CA)13 repeat,^29^ were exposed to activated PMNs for 24 h, and then expanded for 7 days. Analysis of the fluorescent fraction by flow cytometry revealed a significant rise in the number of transiently mutated (M1) fraction whereas the increase in the highly fluorescent population (M2) was not significant (fig 6). No changes in the mutant fraction were observed with non-activated PMNs. This result could suggest that the PMN-induced G2/M arrest is a consequence of increased replication errors. As PMNs did not increase the number of permanent mutations (M2 cells), it is likely that the DNA repair system was functional.

**Figure 7:**
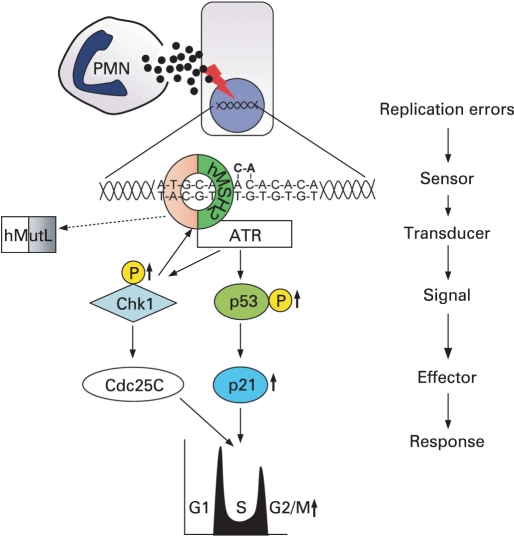
Model of polymorphonuclear (PMN)-induced G2/M cell cycle arrest in colon epithelial cells. An oxidative burst of activated PMNs releases a variety of genotoxic factors [including, but not limited to reactive oxygen species (ROS)] which lead to replication errors [in our system a frameshift at a (CA)13 microsatellite] followed by a G2/M cell cycle arrest. The G2/M checkpoint prevents cells from initiating mitosis when they experience DNA damage. This pathway relies on the presence of functional hMSH2 which is an essential component of the hMutS heterodimer (that also includes hMSH3 or hMSH6; depicted in orange). The hMutS heterodimer acts as a sensor of replication errors and may initiate mismatch repair (by recruiting the hMutL complex). hMSH2 is capable of physically interacting with ATR forming a signalling module. Next, the checkpoint kinase Chk1 undergoes ATR-mediated phosphorylation. Active Chk1 phosphorylates Cdc25c which is inactivated and cells are halted at G2/M. ATR also mediates the phosphorylation of the tumour suppressor p53 (at site serine 15) causing accumulation of the cyclin-dependent kinase inhibitor p21 and cell cycle arrest. The presence of hMSH2, p53 and p21 are essential for the PMN-driven installation of this G2/M checkpoint, a pathway that acts independently of hMLH1 (which is needed for mismatch repair).

## DISCUSSION

Ulcerative colitis is a chronic inflammatory disease of the large intestine which is associated with increased CRC risk.^1^ ^45^ The mucosal injury of active ulcerative colitis is characterised by enhanced transepithelial migration of activated PMNs forming crypt abscesses.^3^ In this study, we have developed an in vitro co-culture model in which primary PMNs acted as effectors and colon cell lines as targets of inflammation-driven carcinogenesis. In our study colon cells responded to activated PMNs or granulocyte-like HL60 cells by slowing proliferation and arresting in the G2/M phase of the cell cycle (fig 2A,B). This observation is consistent with previous reports in which exposure of colon epithelial cells to H_2_O_2_ and macrophages lead to G2/M arrest.^4^ ^28^

Cellular damage induces responses that enable the organism either to eliminate or cope with the damage. DNA damage response reactions include removal of damaged DNA and restoration of the continuity of the DNA structure; activation of a DNA damage checkpoint, which arrests cell cycle progression in order to allow repair and transmission of damaged or incompletely replicated chromosomes; or apoptosis, which eliminates seriously damaged cells.^39^ In most of the cell lines analysed in this study, exposure to activated PMNs caused an arrest of the cell cycle in G2/M, consistent with the activation of a post-replication DNA-damage checkpoint. Evidence for the installation of such a checkpoint,^46^ apart from the G2/M arrest (fig 2A), includes phosphorylation of Chk1 at the ATM and ATR target sites Ser317 (fig 3A) and Ser345 (data not shown);^47^ phosphorylation of the p53 tumour suppressor protein at site Ser15 (fig 3A,C);^48^ increased expression of the cyclin-dependent kinase inhibitor p21^waf1/cip1^ (fig 3A,C); phosphorylation of the histone isoform γ-H2AX at Ser 139 (data not shown) and cleavage of caspase-7 (data not shown).

p53 is essential for the maintenance of a G2/M arrest following oxidative stress. In fact, p53 contributes to the inhibition of cdc2, the mitotic cyclin-dependent kinase through Gadd45, p21^waf1/cip1^, and 14-3-3σ. Cyclin B1 is required for cdc2 activity, and repression of the cyclin B1 gene by p53 also contributes to blocking entry into mitosis.^49^ After disruption of either the p53 or the p21^waf1/cip1^ gene, gamma-irradiated cells progressed into mitosis in spite of extensive damage.^30^ In our system, p21^waf1/cip1^ expression and p53 phosphorylation increased upon exposure to activated PMNs and cells lacking p53 or p21^waf1/cip1^ failed to undergo a G2/M arrest (fig 4), suggesting that the p53 pathway is required for the response to PMN-induced damage. However, although SOD and CAT scavenge ·O_2_^−^ and H_2_O_2_ (fig 5A), and completely reverse the H_2_O_2_ -induced G2/M arrest (fig 5C), they only partially reduced the PMN-induced phosphorylation of p53 (fig 5B) and were unable to reverse the cell cycle arrest caused by PMNs (fig 5C), suggesting that activated PMNs also release additional molecules which can induce G2/M arrest. Another potentially harmful species released by PMNs are chlorinating agents (containing an active chloride in a formal +1 oxidation state, eg, HOCl).^50^ However, the HOCl-specific scavenger taurine^51^ did not compensate the PMN-induced G2/M arrest (data not shown). Further limitations of the model presented are the use of colon cancer cell lines instead of primary colon epithelial cells as well as freshly isolated PMNs which vary from volunteer to volunteer in their composition of released products. Another limitation is the effector:target ratio and time of exposure of colon epithelial cells in our co-culture system as the number of PMNs infiltrating the colon mucosa and the duration of the inflammatory stress are far more dramatic in ulcerative colitis. Direct contact of PMNs with epithelial cells may further increase the observed effects, but is difficult to standardise in vitro.

Eukaryotic MMR include two different heterodimeric complexes of MutS-related proteins: hMutS-α (hMSH2/hMSH6) and hMutS-β (hMSH2/hMSH3). These complexes have different mispair recognition properties and abilities to support MMR. hMutS-α senses single-base mismatches and small insertion–deletion loops, whereas hMutS-β recognise insertion–deletion loops.^17^ Our results suggest that the hMutS-α or hMutS-β heterodimer may be responsible for the response induced by oxidative stress during inflammation (fig 2A). In fact, cells lacking hMSH2 (Lovo colon cells and HEC59 endometrial adenocarcinoma cells), but not their nearly isogenic counterparts, in which an extra chromosome 2 has been reintroduced, failed to arrest in G2/M upon co-culture with activated PMNs (fig 2A). As shown in fig 3A, the phosphorylation of Chk1 and p53 was almost absent in Lovo cells after 8 h. In comparison, Lovo+chr2 cells responded much stronger to PMN induced stress. Indeed, it has been reported that hMSH2 physically interacts with Chk1 and Chk2,^35^ as well as with DNA damage sensors Rad51 and Mre11,^52^ and that this interaction is required for the installation of cell cycle arrest. However, we can not exclude the possibility that the hMSH2 partners hMSH6 and hMSH3 are also needed for the installation of G2/M arrest. Interestingly, when H_2_O_2_ was used in control experiments, the G2/M arrest was also achieved in cells lacking hMSH2 (data not shown).

The DNA damage signalling, induced by activated PMNs, seemed to be more pronounced in HCT116+chr3 cells (fig 3A). Here, in fact, phosphorylation of p53 and Chk1 was already visible upon 8 h exposure to activated PMNs (fig 3A), whereas HCT116 showed very low levels of phosphorylation, which increased at 24 h (fig 3C). HCT116+chr3 express wild-type hMLH1, but also other genes that are present on the extra chromosome 3. An interaction between hMHL1 and the checkpoint sensor components hMre11^40^ and Nbs1^53^ has been reported as well as the co-localisation of MMR proteins and the MRN (Mre11-Rad50-Nbs1) complex to foci of DNA damage.^53^ However, in our study, HCT116-mlh1-2 cells, expressing almost physiological levels of wild-type hMLH1, have a response more similar to that of the parental HCT116 cells than to HCT116+chr3, suggesting a marginal role for hMLH1 in the response of colon cells to activated PMNs. A number of other proteins have been implicated in MMR, including DNA polymerase delta, single-strand binding protein RPA, clamp PCNA, clamp loader replication factor C (RFC), exonuclease 1 (EXO1), endonuclease FEN1,^13^ as well as ATR; all these proteins are involved in synthesising DNA and associated with the replication fork.^54^ We may speculate that the accelerated activation of Chk1 and p53 phosphorylation observed in the HCT116+chr3 upon co-culture with activated PMNs may be a consequence of the introduction of an extra copy of the wild-type ATR gene, which is located on chromosome 3. However, since exposure to ultraviolet light strongly activates this DNA damage pathway in all investigated cell lines (fig 3A) this effect seems to be specific for the exposure to activated PMNs.

In ulcerative colitis, p53 and replication errors at repetitive CA sequences are occurring at an early stage within chronic inflamed mucosa independent of dysplasia.^55^ ^56^ Although the risk of CRC development in ulcerative colitis is increased, the high frequency of these mutations (around 40% of patients) should actually result in far more cancers. One explanation would be that these mutations are of transient nature only. In fact, longitudinal studies of CA repeats on chromosomes 5 (D5S346) and 17 (D17S250) show reversions of mutations over time.^57^ In our model, PMNs increased the number of replication errors in HCT116+chr3 A3.1 and A3.7 cells (fig 6). However, the observed mutations were more of a transient nature. They are a fingerprint of a PMN-induced increase in replication errors, which have initiated the hMSH2-dependent activation of the G2/M checkpoint. Thus, at its current stage our model reflects the ability of epithelial cells to recover from or counteract PMN-induced stress rather than to accumulate mutations, which may finally lead to cancer.
